# Development and Initial Outcomes of the Interdisciplinary ‘Early Identification and Intervention for Infants Network’ (Ei3) in Los Angeles

**DOI:** 10.3390/jcm13237442

**Published:** 2024-12-06

**Authors:** Christiana D. Butera, Amy Yeh, Manoj Biniwale, Edward Bloch, Debi Craddock, Mary Doyle, Sai N. Iyer, Kari S. Kretch, Nora Liu, Christine B. Mirzaian, Barbara Sargent, Priscilla Solano, Margaret Swaine, Stacey C. Dusing

**Affiliations:** 1 Division of Biokinesiology and Physical Therapy, Herman Ostrow School of Dentistry, University of Southern California, Los Angeles, CA 90033, USA; kretch@usc.edu (K.S.K.); bsargent@pt.usc.edu (B.S.); stacey.dusing@usc.edu (S.C.D.); 2 Los Angeles General Medical Center, Los Angeles, CA 90033, USA; amyyeh@usc.edu; 3 Keck School of Medicine, University of Southern California, Los Angeles, CA 90033, USA; biniwale@usc.edu (M.B.); cmirzaian@chla.usc.edu (C.B.M.); 4 Cedars-Sinai Medical Center, Los Angeles, CA 90048, USA; 5 Los Angeles County Department of Public Health, El Monte, CA 91731, USA; ebloch@ph.lacounty.gov (E.B.); nliu@ph.lacounty.gov (N.L.); 6 Los Angeles County California Children’s Services, El Monte, CA 91731, USA; dcraddock@ph.lacounty.gov (D.C.); mdoylemd@gmail.com (M.D.); 7 Department of Pediatrics, University of California Los Angeles, Los Angeles, CA 90095, USA; siyer@mednet.ucla.edu; 8 Children’s Hospital Los Angeles, Los Angeles, CA 90027, USA; 9 Eastern Los Angeles Regional Center, Alhambra, CA 91803, USA; psolano@elarc.org; 10 North Los Angeles County Regional Center, Chatsworth, CA 91311, USA; mswaine@nlacrc.org

**Keywords:** early detection guidelines, cerebral palsy, interdisciplinary team formation, Hammersmith Infant Neurologic Examination, capacity building, workforce training, implementation, stakeholder engagement

## Abstract

**Background/Objectives**: The Early Identification and Intervention for Infants (Ei3) Network is an interdisciplinary team dedicated to improving early detection and intervention of cerebral palsy (CP) in California. This paper describes the key (1) awareness-building and (2) capacity-building strategies utilized by the Ei3 Network in the first two years. **Methods**: Awareness-building methods included interactive conference discussions, resource deliverable creation, and the creation of a framework for dissemination. Capacity-building methods were hosting assessment training, gathering stakeholder feedback, and implementation training. All deliverables were created with a minimum of 3 review and revision cycles. We planned, hosted, and provided scholarships for training, including the Hammersmith Infant Neurological Examination (HINE), Prechtl’s GMA, and an implementation conference. Preliminary descriptive statistics and paired samples *t*-tests were performed on HINE training surveys. **Results**: Seven resource deliverables were created and distributed. A professional website, @steps2home.org, was launched. Online channels gained followers (146, Instagram; 198, X; 298, Mailchimp). Providers participated in various trainings: the Sykes Symposium on early detection and intervention (70), HINE (211), GMA Basic or Advanced (46), and Implementation Conference (six facilities from April 2023 to June 2024). HINE training participants reported increased ability to identify CP (*p* = 0.001), knowledge of (*p* = 0.004) early detection, ability to implement early detection guidelines (*p* < 0.001), and confidence in performing the HINE on the post-test (79.87%). **Conclusions:** The first two years of Ei3 resulted in increased awareness of the early detection guidelines through resources to help providers and families navigate complex care systems and dissemination through online and in-person strategies. We built capacity by training an increased number of providers to identify infants at risk of CP and implement the early detection guidelines. Engagement of stakeholders in focus groups, reviewing documents, and including a parent panel throughout the process increased the value of this work and should support the expansion of the network in the next year.

## 1. Introduction

Cerebral palsy (CP) is the most common physical disability in childhood, affecting approximately 1.6–4 per 1000 children worldwide [1]. CP is a neuromotor condition impacting movement, muscle tone, and posture. It results from a malformation or lesion in the developing brain [1,2,3,4]. In addition to motor impairments, co-occurring impairments in sensation, perception, cognition, communication, and behavior, as well as a range of secondary conditions, may all accompany CP [1,4,5]. Managing CP over the lifespan starts in infancy, and systems of care are evolving to improve service delivery and health outcomes. 

Identifying a community-based need: Early diagnosis and CP-specific early intervention are essential for maximizing the developmental trajectories of children with CP [6]. In 2017, international guidelines were published recommending specific timing and standardization of diagnostic measurement tools for the early accurate detection of CP. Implementation of the early detection guidelines allows for infants as young as 3 months of age to be diagnosed with or “at high risk of” CP [6]. The implementation of these early detection guidelines throughout the world has resulted in a reduced age of CP diagnosis and alters the life course of infants with early life adversity [7]. While these developments are encouraging, the United States has been slow to implement the early detection guidelines, with few studies reporting implementation findings [8,9]. A lack of coordination across sites, lack of provider training on recommended assessment tools, and differing regulations and definitions across states and counties in the United States may result in these advances in early diagnosis not being universally implemented. 

In 2018, a network of hospitals, which locally included the University of California, Los Angeles (UCLA), was funded by the Cerebral Palsy Foundation to build capacity for implementing the international guidelines for the early detection of CP [9]. In 2022, the Division of Biokinesiology and Physical Therapy at the University of Southern California (USC) hosted the Sykes Symposium conference on the implementation of the early detection guidelines, including training in the General Movement Assessment (GMA), a crucial measure needed for implementation. The discussion and brainstorming sessions built into the symposium identified several knowledge gaps that needed to be addressed to increase the success of implementation locally (Table 1).

Developing the Ei3 Network: With several gaps and challenges remaining in Los Angeles (LA), and with funding from the USC Zumberge Research Coordination & Team Building Award, we formed the Early Identification and Intervention for Infants (Ei3) Network. Based on surveying relevant partners in the early detection and intervention landscape, the following types of partners and organizations were invited to be involved: research scientists, community members (parents/caregivers), High-Risk Infant Follow-Up Clinics (physicians, therapists), Regional Centers (monitored by the Department of Developmental Services which implements California Early Start, California’s early intervention program for infants and toddlers with or at risk for a developmental disability, therapists, service providers and case managers), Neonatal Intensive Care Units (NICU, physicians, therapists), and Los Angeles County California Children’s Services (CCS) program (Title V program providing diagnostic and treatment services, medical case management, and physical and occupational therapy services to children under age 21 years with CCS-eligible medical conditions, including CP) representatives. 

Based on the criteria above, the Ei3 Network was formed with an interdisciplinary team of key partners and organizations who expressed interest in the implementation of the early detection guidelines for CP in Los Angeles. Four smaller focused subgroups within Ei3 were each designed to target a specific goal: (1) Roles and Responsibilities, (2) Policy Clarifications, (3) Discharge Planning and Intake, and (4) Community (Table 2). The full Ei3 network met monthly as a whole team, with subgroups meeting weekly as needed. 

Aim of the Project: The Ei3 network’s aims were to increase awareness of the early detection guidelines for CP, build capacity for implementation, and garner community support for implementation in Los Angeles.

The objectives of this paper are to describe (1) the key awareness-building strategies (resource and deliverable creation, building a framework for dissemination) and (2) the key capacity-building strategies (hosting assessment training, gathering stakeholder feedback, and implementation training) utilized by the Ei3 Network in the first two years, with a specific focus on the impacts and outcomes on relevant stakeholders. Throughout the paper, we use the word “stakeholder” to mean a community partner who has a vested interest in the work in their own life and is affected by or responsible for health-related decisions. For our purposes, depending on which resource we are reviewing, this includes individuals with developmental disabilities, caregivers of children with developmental disabilities, or clinical practitioners who work with families with disabilities.

## 2. Materials and Methods

**Figure 1:**
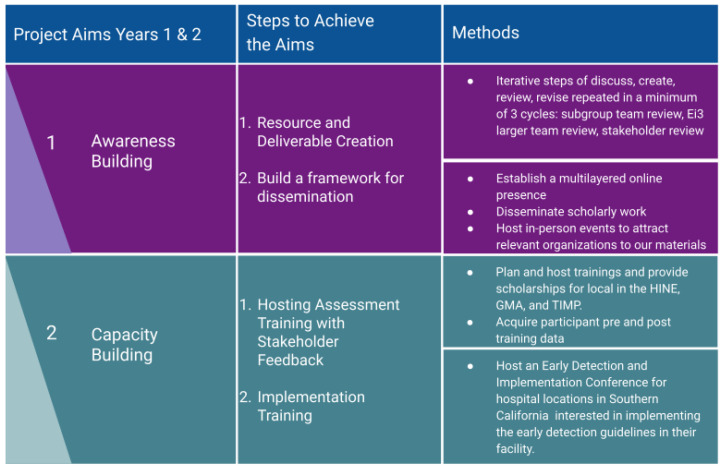
Aims and Methods Overview.

The resulting team and their roles can be found in Table 2. An overview of the aims, action steps, and methods can be found in Figure 1.

### 2.1. Awareness Building 

**Resource and Deliverable Creation.** One key goal of the formation of Ei3 was the development of several resources for caregivers, clinicians, and policymakers that could be used to build awareness of the early detection guidelines. This initiative was led by the Ei3 Roles and Responsibilities subgroup, which focused on educating both providers and community members by creating resource handouts on current decision-making to determine who in the healthcare system was responsible for supporting each aspect of care.

The Policy Clarifications Group reviewed legislation that specifies California’s regulatory definition of CP and criteria to qualify for the CCS Medical Therapy Program (MTP) with a diagnosis of CP (California Code of Regulations Title 22, § 41517.3(a)(2) and § 41517.5). The Policy Clarifications Group also reviewed the current policy governing High-Risk Infant Follow-Up (HRIF) Program Services (DHCS Numbered Letter 05-1016) and whether early detection guidelines could be implemented within current policy parameters. The planned deliverables were (1) a summary document on CCS MTP medical eligibility criteria and (2) a document for medical and therapy providers suggesting how to complete assessments consistent with early detection of CP guidelines while following the current HRIF policy.

The Discharge Planning group was tasked with working with NICU, HRIF, and CCS physicians to increase the clarity of NICU discharge summaries, including key early detection metrics that may be completed by therapists or primary care providers (physicians, physician assistants, nurse practitioners) and information needed by the Regional Centers and Los Angeles County CCS to expedite eligibility determination and the start of care. A lay language version of early detection assessment interpretation was also created to support caregivers’ advocacy for their infants’ eligibility. The Community group aimed to develop 2 handouts that provide information to families of high-risk infants. The first would include information on which contributing factors placed their child at higher risk for developing CP and the signs or symptoms that warrant additional assessment in the first year of life. The second caregiver handout would provide key descriptions of the services available through HRIF, CCS, and Regional Centers, as well as information on how to access care. 

All deliverables were created using an iterative approach with several steps and cycles, including Ei3 members, advisory board, and stakeholder review for quality improvement. The steps followed a pattern of discuss, create, review, and revise. Step 1 was to develop the materials with the feedback of team experts and stakeholders. Step 2 was to create a deliverable based on those discussions. Once the handouts were made, they were reviewed, and feedback was provided for step 3. Lastly, step 4 was that materials were revised based on reviewer feedback. There were a minimum of 3 cycles of review and revision completed per deliverable, which included (1) a subgroup team review cycle, (2) a larger full Ei3 team review cycle, and (3) a stakeholder review cycle (depending on which deliverable, this was either a parent advisory board or working providers like therapists or physicians who provided feedback). Once all materials had been updated throughout several cycles, caregiver-facing documents were translated into Spanish, made aesthetically pleasing, and given Ei3 branded logos and QR codes. 

**Building a Framework for Dissemination.** After the creation of resources and deliverables, it was crucial to have a plan in place for how the public would be able to access this information and how to distribute it widely. In order to develop a dissemination strategy for our community of providers and families, we aimed to establish a multilayered online presence and to have a physical and written presence in the academic community. This online presence would include branding and logo identity for the Ei3 network, the design, development, and launch of a website, the creation of several social media handles, the creation of content to populate these channels, as well as a list-serv for anyone who wanted to join our community and receive newsletters, training updates, or resources via Mailchimp campaigns. The academic dissemination would include launch tables at clinical training, publication of the capacity-building process, and attending and presenting at an academic conference. 

Using funds from the initial capacity building grant, we completed the following steps to create our website (steps2home.org): (1) hired a web designer for branding, logo creation, and site layout and design, (2) purchased a web address and selected a web hosting platform, (3) created all images, copy, new stories, and structure of content to populate the site, (4) hired a web development team to build the site, (5) tested the site for bugs and changes, (6) created a Mailchimp account to engage families to subscribe and sign up for updates from our network that could be integrated directly into the WordPress site. Additional social media channels were created to link and drive traffic to the website, as well as to reach a wider audience. We created Instagram, Facebook, X, and YouTube channels (all handles are @steps2homebyEi3) and worked to create content for all networking platforms. Members of the Ei3 team and parent advisory board were interviewed to create a professional introductory video of our team and our mission, which was added to our YouTube channel and website and was played at all training events (see https://www.youtube.com/watch?v=gdoPxyGrh5k, accessed on 1 October 2024). 

Another aspect of dissemination was hosting in-person events that could attract relevant partners and organizations to our materials while building early detection and intervention capacity. In collaboration with the Sykes Family Chair of Pediatric Physical Therapy, Health and Development at USC and the Cerebral Palsy Foundation, Ei3 team members assisted in planning the 2024 Sykes Symposium. The Sykes Symposium is a biannual conference that supports the translation of new evidence into clinical rehabilitation and medical care to improve the care of children with developmental disabilities. Invitations were spread widely using social media advertisements, university and lab-affiliated email blasts, and contacting wide networks of clinical and research collaborators across the world for these events. 

### 2.2. Capacity Building

One barrier to the implementation of the early detection guidelines is the need for widespread training of all therapists and physicians on specific assessment tools that are necessary for an early diagnosis of CP. To address this gap, we planned and hosted trainings and provided scholarships for local care providers to learn the required assessments in the early detection guidelines, including the Hammersmith Infant Neurological Examination (HINE) [10,11,12] and the Prechtl’s GMA [13]. Training on the Test of Infant Motor Performance (TIMP) [14] was also offered for therapists striving to bridge assessment from the NICU to HRIF.

**Hosting Assessment Training.** One of the Ei3 founders (Dusing) is a certified HINE trainer and was able to provide multiple free HINE training courses offered to several hospitals, local CCS providers, and therapy clinicians across Los Angeles County with organizational help from Ei3 team members for participants and event coordination. Recruitment for training was performed through email contact of individuals who attended the 2022 Sykes Symposium and word of mouth. GMA basic and advanced training was offered immediately following the 2024 Sykes Symposium, and scholarships were offered to local teams that demonstrated an emerging implementation plan. 

**Stakeholder Feedback.** Using a HIPPA compliant Research Electronic Data Capture (REDCap) survey, we acquired de-identified participant pre and post-training data at the HINE training to capture how effective the training sessions were at both teaching the HINE and fostering interest in the importance of early detection. The survey questions helped us better understand if our training was successful at improving awareness of early detection and how well it contributed to building workforce capacity for individuals to confidently administer an early detection assessment. Participants were provided with a QR code and had the option of completing the surveys before and after training, but it was not a required part of the training (IRB UP-24-00248). Basic descriptive statistics and paired samples *t*-tests were performed in SPSS Version 29 on the survey questions and are reported in the results.

**Implementation Training**: Ei3 also hosted an Early Detection and Implementation Conference, held at USC, offered to hospital locations in Southern California that were interested in implementing the early detection guidelines in their facility. Funding from the grant was used to cover the small costs associated with snacks, water, and physical materials to host these training events.

At the Implementation Conference, hospital teams participated in real-time, team-based implementation initiation planning with the support of a trainer, didactic training, and structured planning opportunities. Each team included a minimum of 3 people from the facility who could work together with support for implementation and had the ability to make some decisions about policy change within their setting of care. Typically, teams included NICU, HRIF physicians, therapists, and administrators.

## 3. Results

### 3.1. Awareness Building 

**Figure 2:**
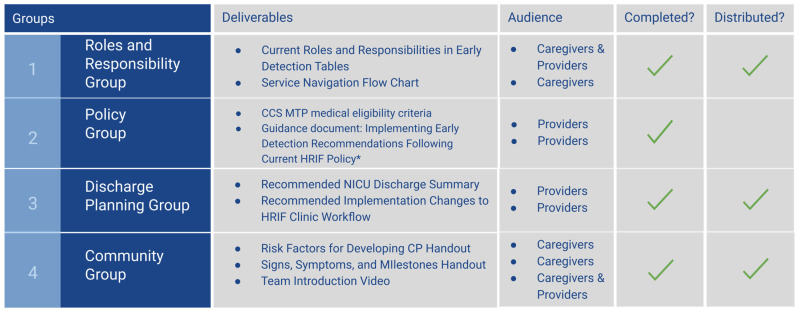
Deliverables Created. * Distribution pending final edits in ongoing discussions.

**Resource and Deliverable Creation.** Following the process listed in the methods, seven core resources were created and distributed by the Ei3 team (see Figure 2 for summary and Appendix A for full materials). The deliverables included a table delineating Current Roles and Responsibilities in Early Detection, a Service Navigation Flow Chart, Recommended Implementation Changes to HRIF Clinic Workflow within the current HRIF policy, Guidance document: Implementing Early Detection Recommendations Following Current HRIF Policy, Recommended new language for a NICU Discharge Summary, Risk Factors for Developing CP Handout, Signs Symptoms and Milestones Handout, and a professional Ei3 Team Introduction Video (Figure 1). After the finalized creation of the caregiver handout, qualitative feedback was collected from seven parents and professionals in a Family Support Specialist Group. The response was largely positive, and the group communicated that they found it useful. The highlighted themes of qualitative responses included appreciating the informative/helpful facts, images, and colors, checkboxes to make the information less dense, and simple caregiver-friendly language. Some critical feedback received was that the caregiver handout should also include numbers to call if parents have concerns, less information for readability, and an example next to the calculator for adjusted age. 

**Figure 3:**
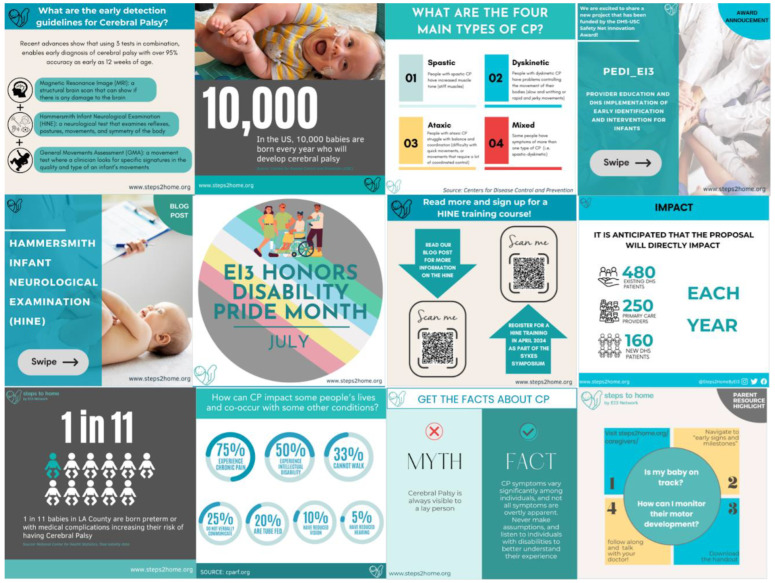
Website and social media content examples.

**Building a Framework for Dissemination.** The online presence dissemination aims included branding and logo identity for the Ei3 network, the design, development, and launch of a website, the creation of several social media handles, the creation of content to populate these channels, digital dissemination of our already created deliverables, as well as a list-serv for anyone who wanted to join our community via Mailchimp Campaigns. We successfully launched a professional website with logo, branding, and blog posts @steps2home.org with a resources page that includes all our deliverables. A Mailchimp account linked to the site has acquired an audience with 298 total subscribers. Email campaigns, including newsletters, training announcements, and resources, have received an average open rate of 50.33% across all communications. We have created and populated social media channels with educational content (Figure 3) and have gained 146 followers on Instagram and 198 followers on X in the past 2 years. 

Another aspect of dissemination was hosting in-person events that could attract relevant stakeholders to our materials. This included launch tables at clinical training, lectures, and attending and presenting at an academic conference. The Sykes Symposium advertising resulted in 70 participants attending. At this event and all other hosted training sessions (see capacity building), all attendees received free physical packets of our deliverables and the option to attend a booth offering business cards with our website information and tablets so people could navigate through our website in person. PI Dusing spoke at the Regional Center Clinician’s Group, which is made up of physicians from 21 Regional Centers throughout California. Lastly, several Ei3 members attended and presented a poster on the process of forming the Ei3 Network at the American Academy for Cerebral Palsy and Developmental Medicine (AACPDM) annual conference in 2023 [15].

### 3.2. Capacity Building 

**Hosting training.** Over the last two years, the Ei3 network has hosted 11 HINE administration and scoring trainings, reaching 211 providers. In addition, 46 people in 2024 and 51 people in 2022 participated in either basic or advanced GMA training. The Implementation Conference also supported step-by-step implementation planning of the early detection guidelines for teams from six facilities across southern California (Los Angeles General Medical Center, UCLA Olive View Medical Center, Children’s Hospital Los Angeles, Miller Children’s Hospital, Rady Children’s Hospital, and Los Angeles County California Children’s Services Medical Therapy Program). The demographic data for all who participated in the pre-test survey at the capacity-building HINE training can be found in Table 3. The sample was primarily made up of female (92.5%) physical therapists (97.4%). The practice setting with the highest number of participants was the Los Angeles County California Children’s Services Medical Therapy Program (51.1%). 

**Figure 4:**
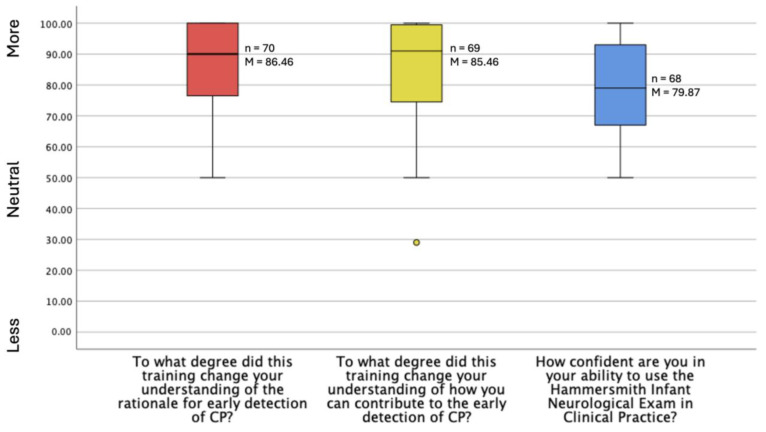
Post-test early detection and HINE outcome.

**Stakeholder Feedback.** The primary goal of this training was to build capacity for early detection implementation by preparing the workforce to administer assessments necessary for early detection. The secondary goal was to increase awareness of early detection methods and their importance among the workforce. From pre-test to post-test, participants reported significantly increased ability to identify CP (*p* = 0.001), knowledge of early detection (*p* < 0.001), as well as increased ability (*p* < 0.001) and knowledge (*p* = 0.004) to implement the early detection guidelines for CP, all with large effect sizes (Table 4). The post-test survey also demonstrated a change in participants’ understanding of the rationale for early detection of CP, how they can contribute to early detection, and confidence in performing the HINE (Figure 4).

## 4. Discussion

Ei3 met the primary goals of the development of this inter-agency and multidisciplinary team: (1) develop a network of inter-agency and multidisciplinary researchers and stakeholders, (2) increase awareness of the early detection guidelines and their principles through resource creation and dissemination network building, (3) build capacity for early detection in Los Angeles by training and educating multiple stakeholders in the system. The first two years of this project resulted in increased awareness of the early detection guidelines through the development of digital and physical resources to help providers and families navigate the complex care systems and dissemination through social media, website, and subscriber following and to improve the accessibility of resources and encourage interaction with the wider community. The capacity-building outcomes were an increase in the number of local providers who are trained to identify children at risk of CP from the earliest time point, as well as six hospitals in Southern California having the tools and a plan for implementation at an individual hospital level. A numbered letter, or document providing administrative policy guidance, was released on 17 October 2024 and will be used for stakeholder training about early detection guidelines. The updated guidance includes language allowing the use of the General Movements Assessment, Hammersmith Infant Neurological Exam, medical risk factor, and neuro-imaging in the determination of eligibility for CCS Medical Therapy Program with a provisional diagnosis of cerebral palsy under 3 years of age (CCS NL: 08-1024).

### 4.1. Limitations

We were unable to collect some pre- and post-Ei3 survey data in the community, which may have given us a better idea of how awareness and capacity had increased. For those who completed surveys as part of the HINE training, it was optional to complete, and, therefore, we had a significant amount of drop-off in the sample for pre- and post-training questions. Further, for those who completed the survey twice, not all questions could be analyzed pre- and post-HINE training due to incomplete responses. These findings should be considered preliminary data.

### 4.2. Future Directions 

Two funding proposals have been awarded in the past 6 months to expand the important work of Ei3. Both projects are directly aligned with the core mission of Ei3 to improve early detection and intervention of CP in California. The first project (Provider Education and Department of Health Services (DHS) Implementation of Early Identification and Intervention for Infants; PEDI-Ei3) will (1) build and provide DHS systemwide evidence-based training to primary care providers about the importance of early identification and referrals, (2) streamline workflow to align with the early detection guidelines of CP at all three DHS hospitals, and (3) increase screening for Adverse Childhood Experiences (ACE) in infants born preterm or with prolonged NICU stay, and increase Referral to Los Angeles County CCS, Regional Centers, and the Enhanced Care Management (ECM) program in High-Risk Infant Follow-Up Clinics. The second project, entitled Building a Southern California Network to Support Early Identification and Intervention for Infants (EI3), will contribute to expanding our capacity-building activities to other regions of California through the following aims: (1) Improve communication of early detection assessment scoring and documentation, (2) expand the training developed for DHS providers to support additional regions of CA, (3) develop a HINE, TIMP, and GMA consulting network where statewide providers can review scoring with experienced examiners to enhance reliability. Lastly, PI Dusing, a certified HINE trainer, is now working with collaborators in the California Perinatal Quality Care Collaborative (CPQCC) in ongoing conversations to integrate the HINE into the list of metrics collected and databased across the state for early detection.

## 5. Conclusions

The Ei3 network achieved the goals of increasing awareness of the early detection guidelines for CP, building capacity for implementation, and garnering community support for implementation in Los Angeles, CA. Preliminary impacts and outcomes on relevant stakeholders were documented through qualitative and quantitative feedback, demonstrating the value of the resources created and the success of their dissemination, as well as an increase in the ability and confidence of care providers to participate in the early detection of CP. The engagement of stakeholders throughout the process increased the value of this training and should support the expansion of the network in the next year. The outcomes of the current study and collaborations outlined in the future directions section highlight the opportunities for this work to impact the implementation and databasing of early detection assessments across the state of California. The successful implementation of the early detection guidelines will allow for earlier and more accurate therapeutic management of infants at risk for CP. The approach of an interdisciplinary and local network can be used in the future as a structure for other groups interested in improving early detection in their local regions.

## Figures and Tables

**Table 1:** Community-based knowledge gaps identified at the 2022 Sykes Symposium.

Topic	Goals	Barriers	Resources Needed
Early Detection	To be open to change A champion organization	Lack of coordination between different systems	Navigators or Navigation systems for families Easier to consume resources on the systems—bridge programs—and case management
	NICUs need to start conversations earlier	CCS MTU qualifications and eligibility is unclear	Collaboration between providers CCS, HRIF, NICU, EI Peer support and mental health
	All providers need to use the same measures Pediatricians need to know more about the GMA and early detection guidelines Help families who are confused about where to go when Common messaging on when diagnosis can be made and by whom	A limited number of therapists have the knowledge and skills to work with infants through 21 y.o.	Policies/systems for remote access Clarification on how early detection guidelines and outcome measures overlap Training on other outcome measures Guide that shows the relationships between CCS eligibility and things measured on the HINE

**Table 2:** Ei3 Network members.

Individual	Job Title/Role(s)	Affiliation(s)	Subgroup(s)
Stacey Dusing PhD, PT, FAPTA	Principal Investigator; Researcher (Associate Professor); Physical Therapist	USC Division of Biokinesiology and Physical Therapy	Roles and Responsibilities Community
Amy Yeh MD, MPH	Principal Investigator; Neonatologist; Assistant Professor of Clinical Pediatrics	Los Angeles General Medical Center; Keck School of Medicine USC	Discharge Planning Policy Clarifications
Christiana Butera PhD, EdM	Assistant Professor of Research	USC Division of Biokinesiology and Physical Therapy	Roles and Responsibilities Community
Kari Kretch PhD, DPT, PT	Researcher (Assistant Professor); Physical Therapist	USC Division of Biokinesiology and Physical Therapy	Community Discharge Planning
Barbara Sargent PhD, PT, PCS	Researcher, Teacher (Associate Professor of Clinical Physical Therapy); Physical Therapist	USC Division of Biokinesiology and Physical Therapy	Roles and Responsibilities Policy Clarifications
Priscilla Solano	Administration and Management (Supervisor of the Early Intervention Department)	Eastern Los Angeles Regional Center *	Roles and Responsibilities Community
Sai N. Iyer MD	Developmental–Behavioral Pediatrician; Health Sciences Assistant Professor; Associate Medical Director High-Risk Infant Follow-up (HRIF) clinic	UCLA Department of Pediatrics	Discharge Planning Policy Clarifications
Mary Doyle MD	General and Medical Therapy Program Medical Consultant	Los Angeles County California Children’s Services **	Roles and Responsibilities Policy Clarifications
Debi Craddock PT, DPT, MS	Medical Therapy Program Physical Therapy Instructor; Physical Therapist	Los Angeles County California Children’s Services **	Roles and Responsibilities Community
Margaret Swaine MD	Medical Services Supervisor; Developmental–Behavioral Pediatrician	North Los Angeles County Regional Center *	Discharge Planning Policy Clarifications
Edward Bloch MD, FAAP	Medical Director, Children’s Medical Services	Los Angeles County Department of Public Health	Discharge Planning Policy Clarifications
Nora Liu PT, DPT, PCS	Therapy Services Chief, Children’s Medical Services; Physical Therapist	Los Angeles County Department of Public Health	Discharge Planning Policy Clarifications
Christine Mirzaian MD, MPH, IBCLC	Attending Physician Pediatrics; Associate Professor of Clinical Pediatrics	Children’s Hospital Los Angeles; Keck School of Medicine USC	Roles and Responsibilities Community
Manoj Biniwale MD	Neonatologist Clinical Professor of Pediatrics (Clinician Educator) Director of NICU Clinical Research	Good Samaritan Hospital Keck Medicine of USC Cedars-Sinai Medical Center	Community Discharge Planning
Scott Gorshein (parent)	Lead—Parent Advisory Group		Reviewer for Caregiver Resources
Anita Bailie (parent)	Lead—Parent Advisory Group		Reviewer for Caregiver Resources

* = Regional Centers are the agencies where California Early Start administers early intervention programs for infants and toddlers with developmental delays or at risk for having a developmental disability and their families. ** = California Children’s Services (CCS) is a Title V state program for children with complex health care needs administered by the California Department of Health Care Services (DHCS). Title V of the Social Security Act provides funding through the Maternal and Child Health (MCH) Block Grant Program. LA County CCS representatives participated as local stakeholders and do not have the authority to act as agency representatives of the statewide CCS program administered by DHCS.

**Table 3:** Demographic data.

Demographic Characteristics		n	Frequency	Valid Percentage
Gender		149		
	Female		135	92.5
	Male		7	4.8
	Nonbinary		1	0.7
	Prefer not to answer		3	2.1
	Not reported		2	
Ethnicity		149		
	Hispanic or Latino		15	10.4
	NOT Hispanic or Latino		123	85.4
	Unknown		6	4.2
	Not reported		5	
Race		149		
	American Indian/Alaska Native		2	1.3
	Asian		46	30.9
	Native Hawaiian/Other Pacific Islander		2	1.3
	Black or African American		5	3.4
	White		84	56.4
	Not reported		10	
Discipline		211		
	Physician—Neonatology		2	1.1
	Physician—Developmental Peds		4	2.3
	Physician—Pediatrics		4	2.3
	Nurse Practitioner		4	2.3
	Physical Therapist		114	64.4
	Occupational Therapist		44	24.9
	Speech Language Pathologist		2	1.1
	Other		3	1.7
	Not Reported		34	
Primary Practice Setting		149		
	Los Angeles, San Diego, or Orange County California Children’s Services Medical Therapy Program		71	51.1
	Los Angeles, San Diego, or Orange County California Children’s Services General Program		2	1.4
	High-Risk Infant Follow-up		6	4.3
	NICU		10	7.2
	In-patient Pediatrics		14	10.1
	Outpatient Therapy Practice		23	16.5
	Outpatient Medical Practice		4	2.9
	Early Intervention		9	6.5
Location		149		
	San Diego County		4	20
	Orange County		1	5
	Los Angeles County		11	55
	Other		4	20
	Not Reported		129	

**Table 4:** Early detection survey feedback.

	Pre-Training			Post-Training			Statistics	
Question	n	M	SD	n	M	SD		
How do you feel about your ability to identify CP? (scale 1–5)	30	3.7	0.837	30	4.37	0.928	**0.001**	1.056
How do you feel about your knowledge of early detection? (scale 1–5)	29	3.34	1.111	29	4.28	0.797	**<0.001**	1.061
How do you feel about your ability to implement early detection in your setting? (scale 1–5)	29	2.86	1.187	29	4.14	0.915	**<0.001**	1.162
How interested are you in implementing the early detection guidelines for CP in your practice? (%)	29	87.86	13.362	29	91.1	11.595	0.029	7.812
How comfortable are you in implementing the early detection guidelines for CP in your practice? (%)	25	53.44	31.095	25	77.44	20.197	**0.004**	38.441
How close are you to fully implementing the early detection of CP in your practice? (%)	21	37	22.72	21	49.38	26.394	0.024	24.234

Note: Boldface text reflects significance according to Bonferroni adjustment based on six comparisons with a significance benchmark of *p* < 0.008.

**Figure A1:**
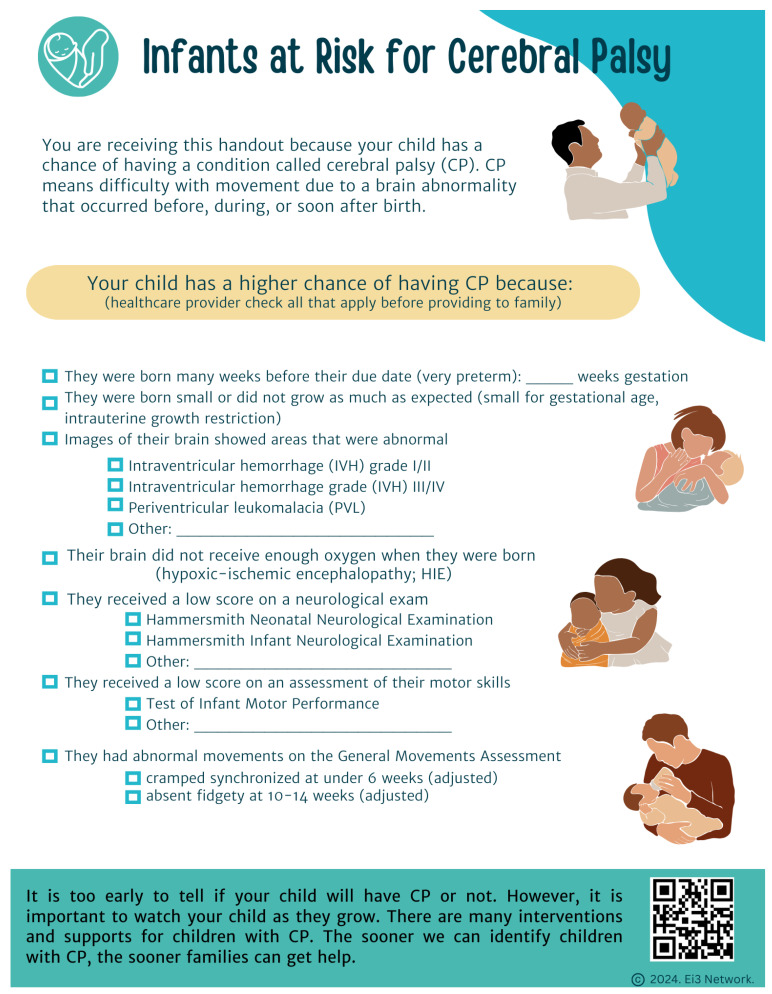
Two-page caregiver handout (English, Page 1).

**Figure A2:**
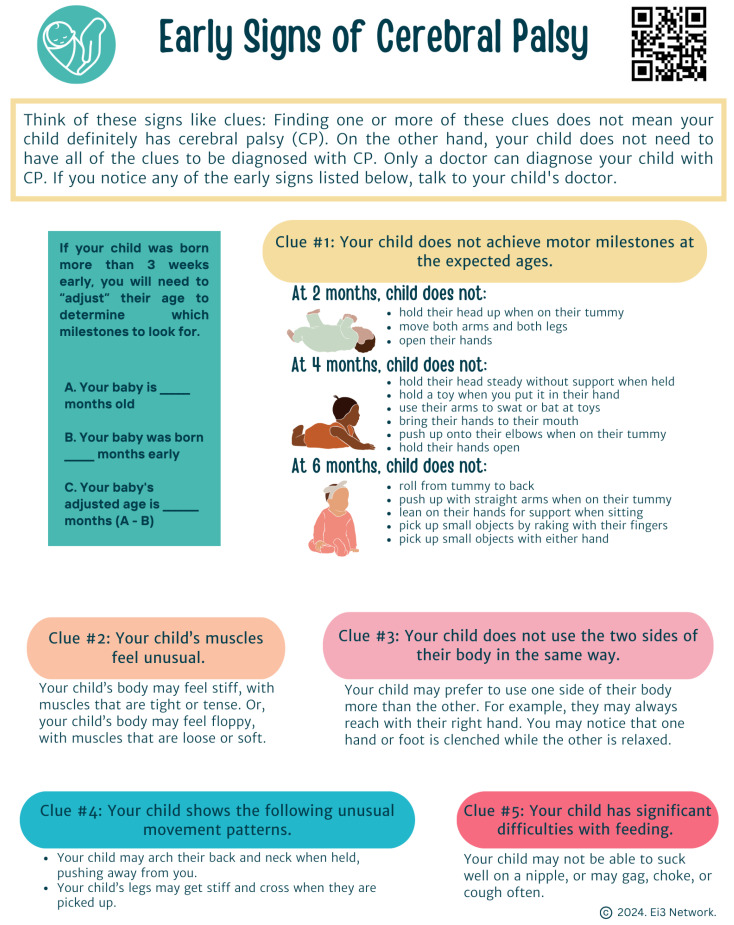
Two-page caregiver handout (English, Page 2).

**Figure A3:**
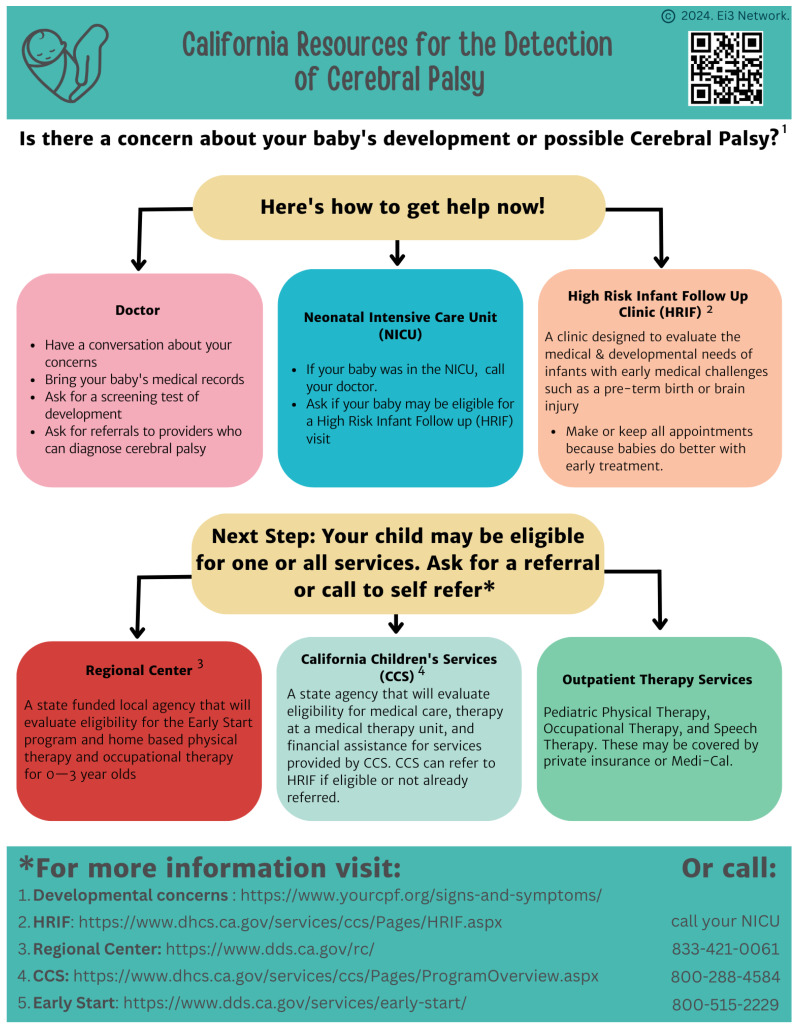
California Resources for the Detection of Cerebral Palsy Handout (English).

**Figure A4:**
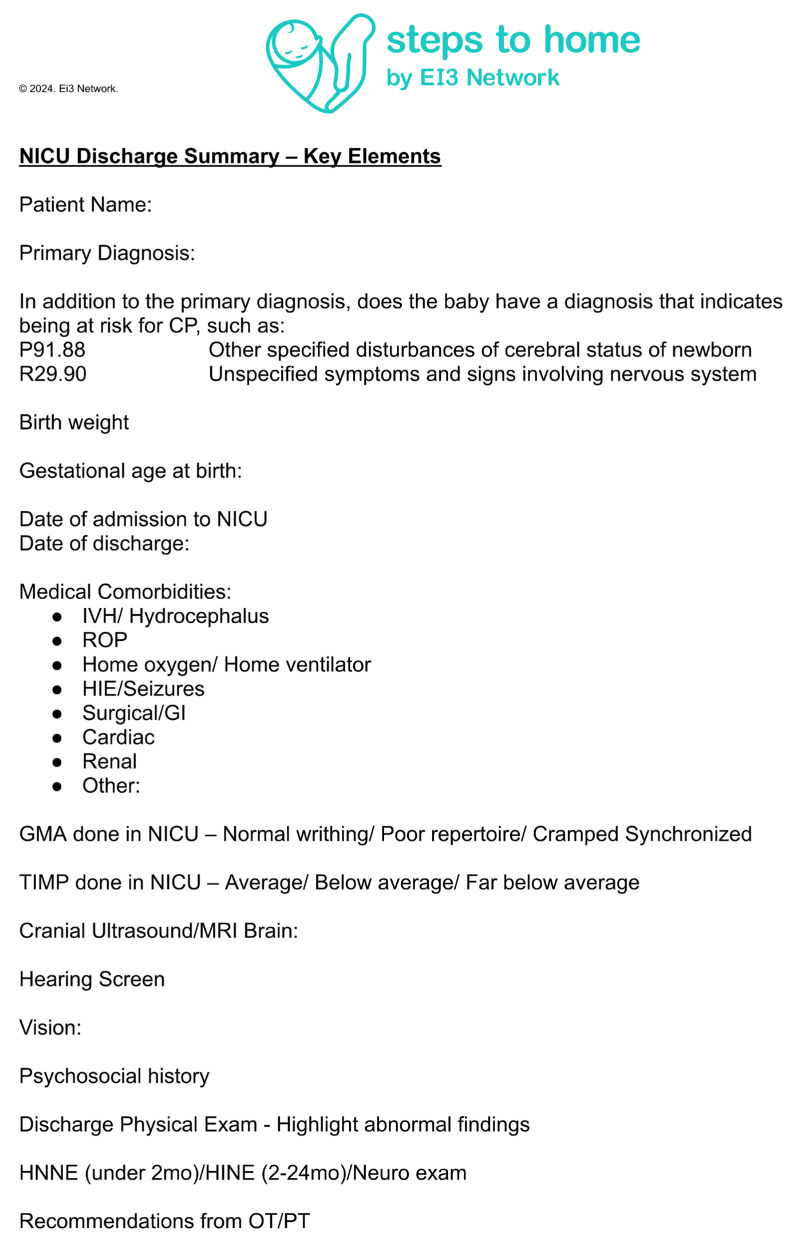

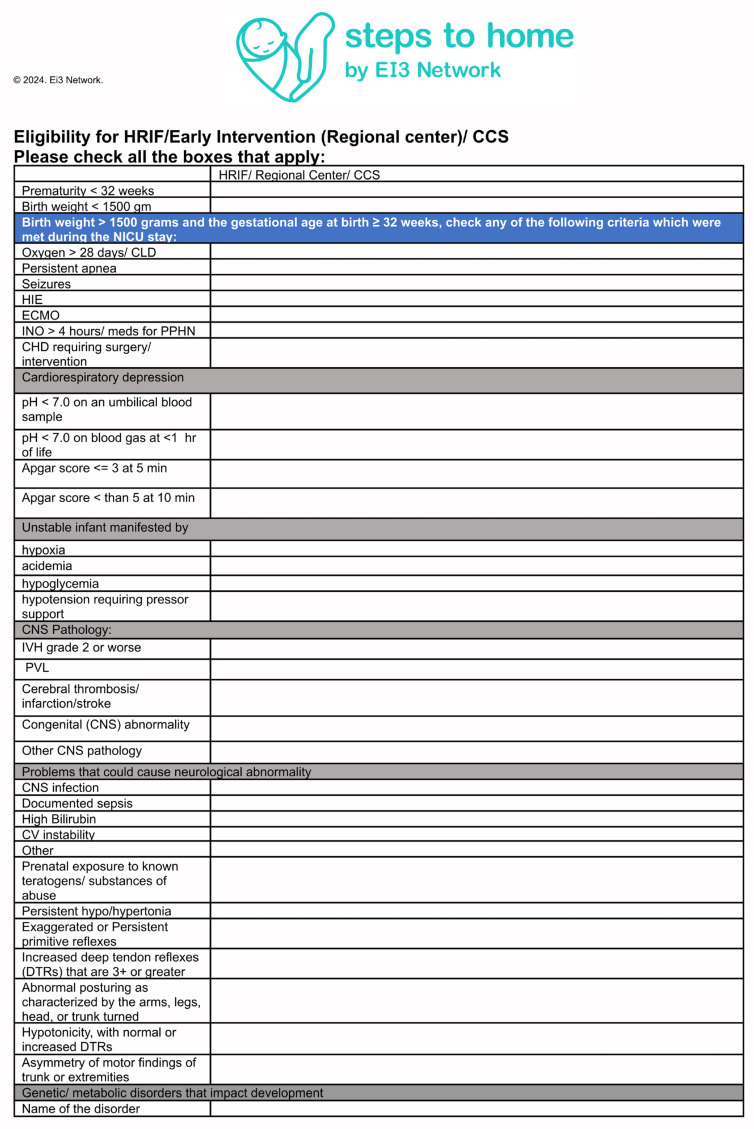
Recommended NICU discharge Summary. CP = Cerebral Palsy; NICU = Neonatal Intensive Care Unit; IVH = intraventricular hemorrhage; ROP = retinopathy of prematurity; GI = gastrointestinal; GMA = General Movements Assessment; TIMP = Test of Infant Motor Performance; MRI = magnetic resonance imaging; HNNE = Hammersmith Neonatal Neurological Examination; HINE = Hammersmith Infant Neurological Examination; OT = occupational therapy; PT = physical therapy; HRIF = High Risk Infant Follow-Up; CCS = California Children’s Services; gm = grams; CLD = chronic lung disease; HIE = hypoxic-ischemic encephalopathy; ECMO = Extracorporeal Membrane Oxygenation; INO = Inhaled nitric oxide; PPHN = persistent pulmonary hypertension of the newborn; CHD = congenital heart disease; pH = potential hydrogen; Apgar = Appearance, Pulse, Grimace, Activity, and Respiration; PVL = periventricular leukomalacia; CNS = central nervous system; CV = craniocervical.

**Figure A5:**
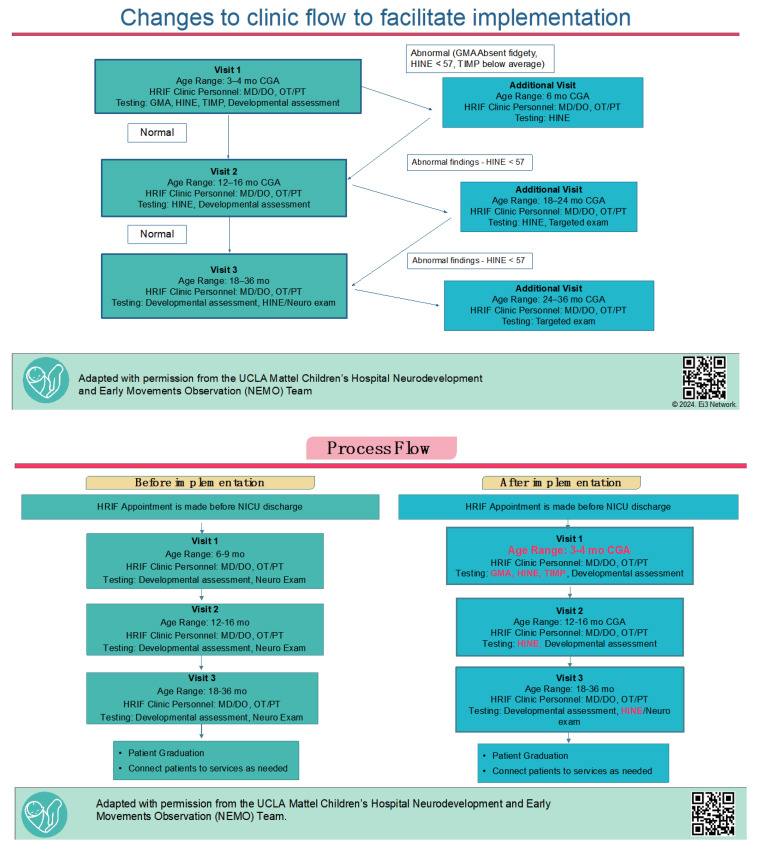
Recommended clinic flow for early detection. mo = months; CGA = corrected gestational age; HRIF = High Risk Infant Follow-Up; MD = medical doctor; DO = doctor of osteopathic medicine; GMA = General Movements Assessment; TIMP = Test of Infant Motor Performance; HINE = Hammersmith Infant Neurological Examination; OT = occupational therapy; PT = physical therapy; CCS = California Children’s Services.

**Figure A6:**
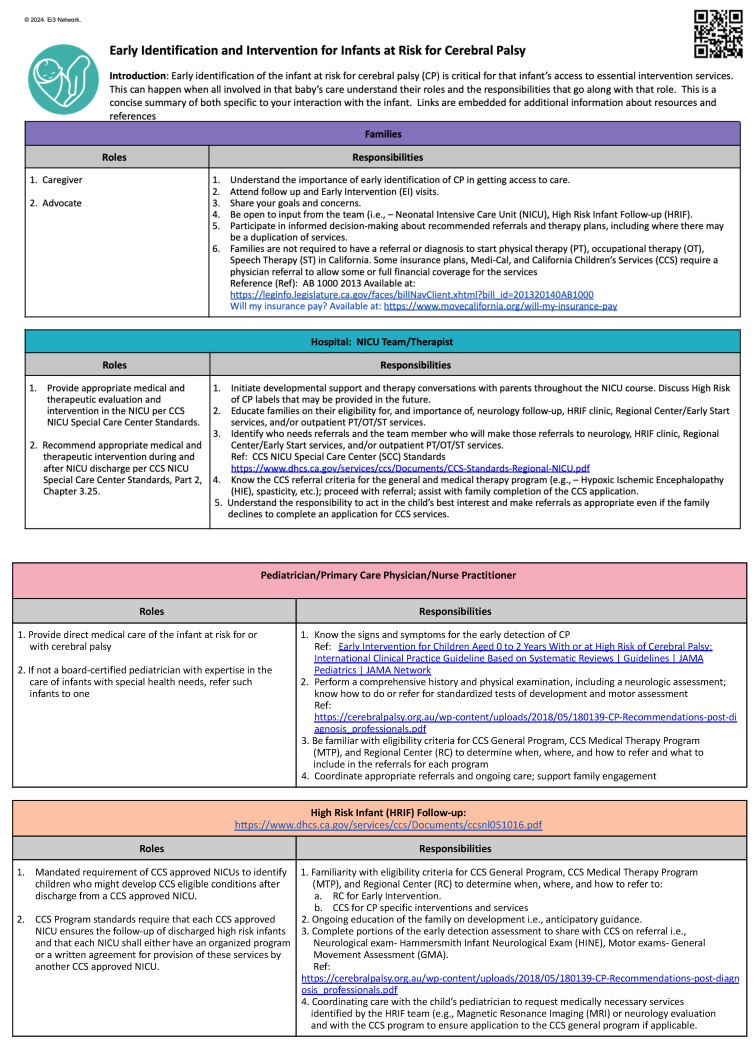

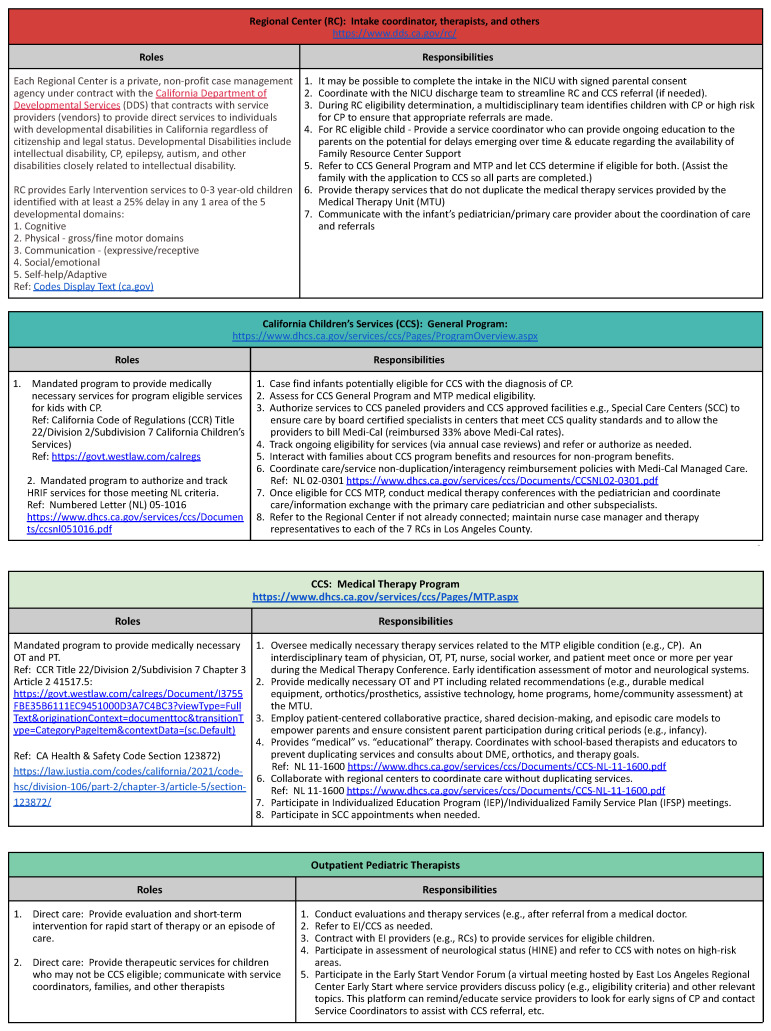

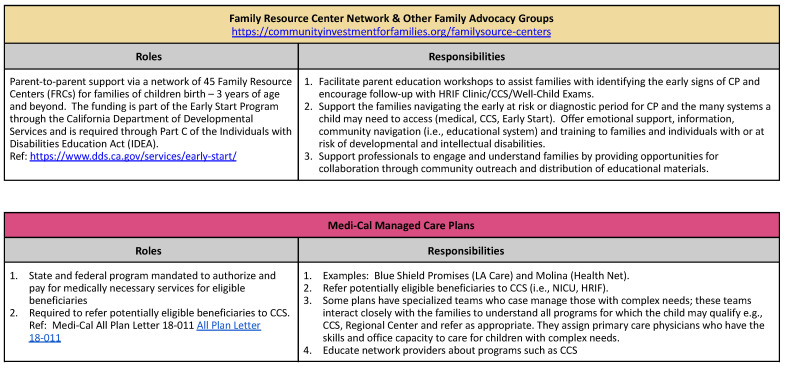
Early Detection Roles and Responsibilities Summary Table.

## Data Availability

The raw data supporting the conclusions of this article will be made available by the authors upon request.

## Glossary of Abbreviations

CCS	California Children’s Services
CCS MTU	California Children’s Services Medical Therapy Unit
CP	Cerebral Palsy
DHCS	California Department of Health Care Services
EI	Early Intervention
Ei3 Network	Early Identification and Intervention for Infants Network
GMA	Prechtal’s General Movements Assessment
HINE	Hammersmith Infant Neurological Examination
HRIF	High-Risk Infant Follow-Up
LA	Los Angeles
MCH	Maternal and Child Health
NICU	Neonatal Intensive Care Unit
TIMP	Test of Infant Motor Performance
UCLA	University of California Los Angeles
USC	University of Southern California
